# Performance validation of deformable image registration in the pelvic region

**DOI:** 10.1093/jrr/rrt045

**Published:** 2013-07

**Authors:** V. Zambrano, H. Furtado, D. Fabri, C. LÜtgendorf-Caucig, J. GÓra, M. Stock, R. Mayer, W. Birkfellner, D. Georg

**Affiliations:** 1 EBG MedAustron GmbH, Wr. Neustadt, Austria; 2 Department of Radiooncology, Medical University of Vienna/AKH Wien, Waehringer Guertel 18–20, 1090 Vienna, Austria; 3 Centre of Medical Physics and Biomedical Engineering, Medical University of Vienna, Waehringer Guertel 18–20/4L, A-1090 Vienna, Austria; 4 Christian Doppler Laboratory for Medical Radiation Research for Radiation Oncology Medical University of Vienna, Waehringer Guertel 18–20, A-1090 Vienna, Austria

**Keywords:** radiotherapy, registration, deformable, organ, adaptive radiotherapy

## Abstract

Patients undergoing radiotherapy will inevitably show anatomical changes during the course of treatment. These can be weight loss, tumour shrinkage, and organ motion or filling changes. For advanced and adaptive radiotherapy (ART) information about anatomical changes must be extracted from repeated images in order to be able to evaluate and manage these changes. Deformable image registration (DIR) is a tool that can be used to efficiently gather information about anatomical changes. The aim of the present study was to evaluate the performance of two DIR methods for automatic organ at risk (OAR) contour propagation. Datasets from ten gynaecological patients having repeated computed tomography (CT) and cone beam computed tomography (CBCT) scans were collected. Contours were delineated on the planning CT and on every repeated scan by an expert clinician. DIR using our in-house developed featurelet-based method and the iPlan^®^ BrainLab treatment planning system software was performed with the planning CT as reference and a selection of repeated scans as the target dataset. The planning CT contours were deformed using the resulting deformation fields and compared to the manually defined contours. Dice's similarity coefficients (*DSC*s) were calculated for each fractional patient scan structure, comparing the volume overlap using DIR with that using rigid registration only. No significant improvement in volume overlap was found after DIR as compared with rigid registration, independent of which image modality or DIR method was used. DIR needs to be further improved in order to facilitate contour propagation in the pelvic region in ART approaches.

## INTRODUCTION

The standard approach for treatment planning in conventional radiation therapy, intensity-modulated radiotherapy (IMRT) or particle beam therapy is based on a single ‘snapshot’ computed tomography (CT) as the basis for treatment planning. It is well known and documented that during the course of treatment the patient's anatomy can change due to organ filling, tumour shrinkage, weight loss, etc. The management of organ motion and organ deformation has become a key aspect in advanced radiation therapy where steep dose gradients are applied, since such anatomical variations may lead to significant changes in the dose delivery to the tumour and to the surrounding healthy tissues with respect to the original and intended treatment plans.

Several groups have studied anatomical changes using both phantoms and clinical data, showing the potential of adaptive treatment radiotherapy (ART) to optimize organ-at-risk (OAR) sparing when using highly conformal precision radiotherapy techniques [1–5]. Adapting the original treatment plan to the given anatomy every day or week can be a very time-consuming approach. Alternatively, plan libraries can be used, or the anatomic changes can simply be monitored during the course of radiotherapy with a certain tolerance level. Irrespective of ART technique, deformable image registration (DIR) [6] is a primary software tool that can be of great importance in evaluating and taking into account the impact of anatomical changes on the treatment plan quality and delivery for any ART approach. Since manual contouring is a very time-consuming task, contour propagation driven by successful DIR can greatly reduce the workload for the radiation oncologist. If dose accumulation is pursued, then reliable and robust DIR is close to prerequisite.

Many different DIR approaches can be found in the literature, the most popular being biological, mesh, elastic, polynomial or other mathematical model-based registration approaches [3, 7–11]. At present, the challenges of most DIR algorithms in medicine are poor computation efficiency, leading to time-consuming calculations, and an inability to discriminate between bones and soft tissue, leading to distortion of the whole anatomy equally.

In this paper we evaluated the performance of our in-house-developed featurelet-based DIR approach using clinical datasets from gynaecological patients, and we benchmarked this evaluation against the iPlan^®^ BrainLab treatment planning system (TPS) DIR software. Our purpose was to assess the accuracy of DIR for contour propagation as a basis for dose accumulation and adaptive planning. For the purpose of this study, CT to CT (intramodal registration), as well as CT to cone beam computed tomography (CBCT) (intermodal registration), was assessed. By comparing the performance of DIR for both a CT and a CBCT scan we were also able to compare the quality of registration obtained when using these different modalities, since CBCT is frequently proposed for ART but has limited soft-tissue contrast, a well-known disadvantage of this imaging modality.

## MATERIALS AND METHODS

### Patient datasets

Datasets were collected from 10 cervical cancer patients undergoing photon-beam therapy (conformal RT or IMRT) at the Medical University Vienna/AKH, Vienna. As part of their treatment, as well as planning CTs, repeated CT and CBCT imaging was performed during the five weeks of external beam therapy. In our evaluation, for each patient the initial planning CT, one repeated CT and one CBCT were considered. The repeated CT and CBCT (see details below) were acquired during the same treatment fraction. The repeated scans utilized in the framework of this study were taken between Weeks 3 and 5 of the treatment course.

In all imaging data sets (planning CT, repeated CT and CBCT) OARs were manually delineated by an expert physician using the Oncentra Masterplan Nucletron^TM^ TPS (v3.2 Nucletron BV, Veenendaal, the Netherlands), where the original treatment plans were created. Only bladder and rectum contours were taken into account in our study. The structure datasets in all image datasets were exported to the iPlan^®^ BrainLab TPS (v.4.5, BrainLab, Feldkirchen, Germany) and converted to multiple binary single-organ volume files by means of an organ extraction algorithm [12] to be used by our software.

### Imaging protocols

The planning and repeated CT images were obtained with a multi-slice CT scanner (Siemens, Erlangen, Germany) in a spiral mode with an intra-slice resolution of 512 × 512 pixels with 0.917 97 × 0.917 97 mm^2^ pixel spacing with a total of 113 slices with 4-mm slice thickness. For the planning CT image acquisition intravenous contrast was used (Japomiro, Bracco, Vienna, Austria, 90 ml), while repeated CT scanning was performed without contrast media. The contrast was used in planning to highlight the left and right iliac arteries, which have lymph nodes in their vicinity that might require irradiation.

The repeated CBCT images (XVI, Elekta, Crawley, UK) were obtained without contrast before each treatment fraction on an Elekta Synergy linear accelerator (LINAC). The CBCT acquisition protocol (120 kV, 649 mA) was optimized for pelvic imaging with a field of view of 42 cm. The CBCT X-ray unit uses a Perkin Elmer XRD amorphous silicon detector with an active surface of 410 × 410 mm^2^ and 1024 × 1024 pixel resolution. The images obtained were interpolated to a resolution of 1 mm^2^, resulting in a volume with an intra-slice resolution of 410 × 410 pixels with 1 × 1 mm^2^ pixel spacing, and a total of 42 slices of 4-mm slice thickness. The detector panel was located 536 mm from the axis of rotation and the source was located at a distance of 1536 mm from the panel. The images were captured at a fixed frame rate of 2.7 Hz. During the 360º rotation the system acquired approximately 650 planar scans, which were used to make a full 3D image.

### Deformable image registration

Our in-house DIR algorithm [13] is based on the method proposed by Söhn *et al*. [14], and we refined the constraints imposed for the final deformation field calculation. The main advantage of this approach was that it was model independent and solely based on voxel intensities, enabling simple and computationally efficient implementation. The algorithm divides both source (moving) and target (fixed) images into sub-volumes, also called featurelets. Each of the featurelets in the source image is independently rigidly registered to its corresponding one in the target image using a 3-degrees-of-freedom (DoF) rigid registration (RR) approach with three translational parameters as a result. The resulting displacement vector was assigned to each featurelet centre. The total vector field describes the transformation from source to target volume. Since the deformation field consists of displacement vectors only for each of the mega-voxel's (featurelet's) centre, an interpolation was performed in order to assign a value to each voxel. The resulting vector field is the output of our algorithm.

The interpolation function used for this study was the Cosine Window Function (1):
(1)
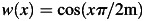



**Fig. 1.**
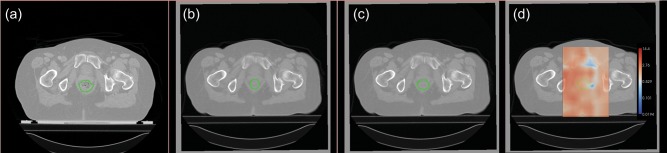
Illustration of the DIR algorithm output for a representative patient: (**a**) axial slice from the planning CT is shown with delineated rectum, (**b**) same axial slice in the repeated CT with manually drawn contour, (**c**) same slice on repeated CT shown with deformed structure, (**d**) resulting deformation vector field when the repeated CT was used as the target image.

Where *m* ε ℕ \ {0} determined the radius of the window or the extent of the kernel for interpolation. This parameter was chosen to be equal to 4. The metric function used for each featurelet RR was Mutual Information [15]. The DIR algorithm iterates for each featurelet until mutual information is maximized, that is, when the best match within each pair of featurelets is found. The user can select an intensity threshold, the 3D gauge of the featurelet grid and the region of the target image where the matching featurelet is searched. We used –2000 for the intensity threshold, 15 × 15 × 15 pixels for the featurelet size, and 25 × 25 × 25 pixels for the search region size in the target image. In addition, a region of interest (ROI) around the pelvic region containing both bladder and rectum was selected for each patient for two reasons: (i) the deformation we were interested to track in our study is completely contained in the selected ROI and (ii) smaller volumes reduce the registration time. Figure 1 shows an example of the featurelet-based DIR algorithm result, where a planning CT with the original rectum contour, a repeated CT with the manually contoured rectum, a repeated CT with the deformed rectum contour, and the deformation vector field is shown.

Our method was benchmarked against the DIR algorithm implemented in the iPlan^®^ BrainLab TPS. This software implementation of DIR uses the Demons method for the deformable registration. In the Demons registration, algorithm object boundaries in the target image are assumed to behave as semi-permeable membranes, while the source image is a grid model diffusing through such membranes [16]. The name of the method comes from Maxwell's demon, through which the behaviour of the aforementioned membranes is explained, even though the analogy with the thermodynamic Maxwell's demon is further improved by introducing attraction. The deformation is, hence, not based on a diffusion model only; it also depends on distance.

In the first step, all repetitive imaging datasets were rigidly registered to the planning CT using the RR tool available in the Oncentra Masterplan Nucletron^TM^ TPS. The overlaps of the original contours on the planning CT and the new contours on the repeated imaging datasets were evaluated (see details below).

In the second step, DIR was performed between the original planning CT and the respective repeated scans. The resulting deformation field was then applied to the original contours on the planning CTs for contour propagation. The final propagated contours were compared to the manually delineated organs on the repeated scans in order to assess the quality of the contour propagation for the DIR algorithm.

### Evaluation of contour agreement

As mentioned above, after DIR the bladder and rectum contours delineated on the planning CT were deformed with the deformation vector field obtained with both the DIR algorithm implemented in our featurelet-based software and in the iPlan^®^ system. The resulting deformed contours for both evaluations were then compared with the manually segmented contours in the respective image dataset. The quality of the deformation was assessed by calculating the volume of the overlap of the resulting contours with the manual contours. The contours were compared by calculating their Dice's similarity coefficients (*DSCs*), which measure the volume overlap percentage (2):
(2)
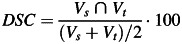

where *V*_*s*_ and *V*_*t*_ are the volumes of the source and the target structures, respectively. Two repeated image-specific *DSC* values were calculated for each fractional patient scan. First, the volume overlap immediately after RR was calculated for the planning CT original contours and the repeated scan manual contours, so as to measure the baseline volume overlap. Second, we calculated the overlap between the planning CT's DIR-deformed contours (propagated contours) and the manual contour (Figure 1).

## RESULTS

Tables 1 and 2 summarize the results obtained for each patient using the featurelet-based method for the inter- and intra-modality DIR approach, respectively. Results are shown as volume overlap percentage (*DSC*) before and after DIR (RR and DIR columns respectively) and *DSC* improvement, which is the difference between the DIR and RR values. The results obtained for Patient No. 10 were not in agreement with the *DSC* values obtained for the other patients. This particular patient exhibited extreme and ‘above average’ anatomical variations and was excluded from our analysis for this reason. A two-tailed Student's *t*-test at 95% CL test showed a *P*-value of *P* < 0.0001 for both image modalities, supporting the decision to exclude the patient from the analysis.

**Table 1.** Summary of the *DSC* results obtained for each patient organ at risk (OAR) after about one month of treatment with the inter-modality approach, using the featurelet-based method to perform DIR

Featurelets	bladder DSC (%)			rectum DSC (%)		
CT- > CBCT	RR	DIR	improvement	RR	DIR	improvement
pt1	82.87	81.36	*−1.51*	89.38	85.03	*−4.35*
pt2	73.79	74.76	0.97	72.88	67.47	*−5.41*
pt3	72.33	73.66	1.33	63.81	60.36	*−3.45*
pt4	23.55	24.86	1.31	44.97	44.33	*−0.64*
pt5	21.80	22.09	0.29	62.56	64.91	2.35
pt6	69.61	67.29	*−2.32*	58.83	60.79	1.96
pt7	76.08	77.61	1.53	61.89	60.83	*−1.06*
pt8	71.33	73.27	1.94	42.17	46.36	4.19
pt9	57.42	56.63	*−0.79*	55.93	57.26	1.33
**mean**	***60.98***	***61.28***	***0.31***	***61.38***	***60.82***	***−0.56***
**std**	***22.72***	***22.55***	***1.50***	***14.13***	***11.94***	***3.31***

The results are sorted by organ (bladder or rectum) and registration type (RR, DIR). The *DSC* improvement is also shown as the difference between the DIR and the RR *DSC* values.

**Table 2.** Summary of the *DSC* results obtained for each patient organ at risk (OAR) after about one month of treatment with the intra-modality approach, using the featurelet-based method to perform DIR

Featurelets	bladder *DSC* (%)			rectum *DSC* (%)		
CT- > CT	RR	DIR	improvement	RR	DIR	improvement
pt1	74.37	77.10	2.73	57.70	61.97	4.27
pt2	38.11	41.62	3.51	43.58	50.02	6.44
pt3	53.67	55.91	2.24	73.40	71.19	*−2.21*
pt4	49.48	50.92	1.44	48.53	47.24	*−1.29*
pt5	51.89	53.13	1.24	73.64	76.52	2.88
pt6	52.15	47.97	*−4.18*	56.47	59.43	2.96
pt7	70.78	75.54	4.76	71.34	71.60	0.26
pt8	77.24	74.22	*−3.02*	70.88	69.08	*−1.80*
pt9	82.00	78.10	*−3.90*	64.04	61.18	*−2.86*
**mean**	***61.08***	***61.61***	***0.54***	***62.18***	***63.14***	***0.96***
**std**	***15.21***	***14.44***	***3.36***	***11.21***	***9.97***	***3.29***

The results are sorted by organ (bladder or rectum) and registration type (RR, DIR). The *DSC* improvement is also shown as the difference between the DIR and the RR *DSC* values.

In both tables, the *DSC* values before DIR vary between patients, indicating different magnitudes of organ deformation. There was no significant improvement after DIR for any of the registrations. The results oscillate between slightly better and slightly worse *DSC* values when compared with the original volume overlap for both organs, indicating that DIR is challenging with these images, that is, that there is not enough information in the images to correctly extract a meaningful deformation field. The maximum improvement was 6.4% in the rectum with CT as the target and an initial *DSC* of 43.6%, and the worst result was –5.4%, also in the rectum, with CBCT as the target and an initial *DSC* of 72.9%. The average improvement in both modalities and for both organs was very close to zero.

The results are very similar for both modalities, showing no statistical difference between the two indicating that either one could be used for the DIR. A two-tailed Student's *t*-test at 95% CL was performed, together with its relative *P*-value, resulting in *t* = 0.26, and *P* = 0.79 for the featurelet, and *t* = 1.07, and *P* = 0.29 for the iPlan^®^.

Tables 3 and 4 summarize the results obtained for each patient using the iPlan^®^ method for the inter- and intra-modality DIR approach, respectively. The tables are organized similarly to the previous ones. For this method there is also no clear trend towards improvement or decrease of volume overlap for the analysed patients. The biggest improvement was of 18.7% in the rectum with CT and an initial *DSC* of 43.6%, the same case as for the best featurelet improvement. The biggest decrease in *DSC* was of –31.2%, also for the rectum, with CBCT and an initial *DSC* value of 89.38. This case can be seen as a failed registration since the initial volume overlap was already quite good. In summary, the average improvement for this method was always within ±5%, depending on the modality, with a greater standard deviation than the featurelet case, indicating a bigger discrepancy of results between patients.

**Table 3.** Summary of the *DSC* results obtained for each patient organ at risk (OAR) after about one month of treatment with the inter-modality approach, using the iPlan^®^ BrainLab TPS method to perform DIR

iPLAN	bladder *DSC* (%)			rectum *DSC* (%)		
CT- > CBCT	RR	DIR	improvement	RR	DIR	improvement
pt1	82.87	77.13	*−5.74*	89.38	58.15	*−31.23*
pt2	73.79	58.59	*−15.20*	72.88	54.11	6.17
pt3	72.33	75.85	3.52	63.81	70.87	7.06
pt4	23.55	27.58	4.03	44.97	47.61	2.64
pt5	21.80	20.63	*−1.17*	62.56	69.99	7.43
pt6	69.61	69.78	0.17	58.83	41.99	*−16.84*
pt7	76.08	77.56	1.48	61.89	73.32	11.43
pt8	71.33	62.25	*−9.08*	42.17	44.57	15.76
pt9	57.42	62.86	5.44	55.93	49.45	*−6.48*
**mean**	***60.98***	***59.14***	***−1.84***	***61.38***	***56.67***	***−4.71***
**std**	***22.72***	***21.09***	***6.87***	***14.13***	***12.05***	***15.12***

The results are sorted by organ (bladder or rectum) and registration type (RR, DIR). The *DSC* improvement is also shown as the difference between the DIR and the RR *DSC* values.

**Table 4.** Summary of the *DSC* results obtained for each patient organ at risk (OAR) after about one month of treatment with the intra-modality approach, using the iPlan^®^ BrainLab TPS method to perform DIR

iPLAN	bladder *DSC* (%)			rectum *DSC* (%)		
CT- > CT	RR	DIR	improvement	RR	DIR	improvement
pt1	74.37	79.06	4.69	57.70	68.89	11.19
pt2	38.11	31.03	*−7.08*	43.58	62.26	18.68
pt3	53.67	61.64	7.97	73.40	67.64	*−5.76*
pt4	49.48	24.33	*−25.15*	48.53	65.37	16.84
pt5	51.89	59.48	7.59	73.64	76.73	3.09
pt6	52.15	58.41	6.26	56.47	59.9	3.43
pt7	70.78	71.29	0.51	71.34	71.53	0.19
pt8	77.24	79.74	2.50	70.88	67.95	*−2.93*
pt9	82.00	76.10	*−5.90*	64.04	62.05	*−1.99*
**mean**	***61.08***	***60.12***	***−0.96***	***62.18***	***66.92***	***4.75***
**std**	***15.21***	***20.18***	***10.60***	***11.21***	***5.24***	***8.82***

The results are sorted by organ (bladder or rectum) and registration type (RR, DIR). The *DSC* improvement is also shown as the difference between the DIR and the RR *DSC* values.

**Fig. 2.**
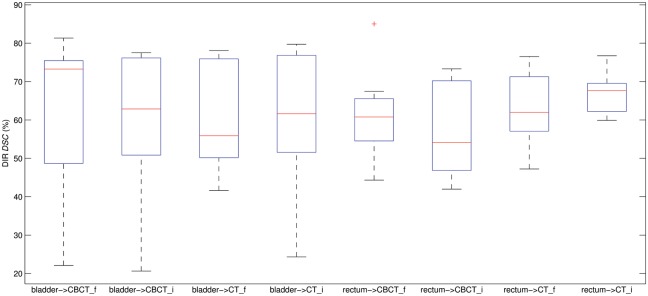
Dice's similarity coefficient (*DSC*) results obtained for rectum and bladder after DIR. From left to right the inter-modality bladder *DSC* for featurelet (f) and iPlan^®^ (i): the intra-modality bladder *DSC* for featurelet (f) and iPlan^®^ (i), the inter-modality rectum *DSC* for the (f) and the iPlan^®^ (i), and the intra-modality rectum *DSC* for the featurelet (f) and the iPlan^®^ (i). Each plot shows the median (central mark, red line), 75th and 25th percentiles (top and bottom of the box), and data extremes (top and bottom whiskers).

Figure 2 shows the plot of the average final *DSC* value for each of the methods in each modality. The results displayed in Fig. 2 clearly indicate that both methods behave similarly, with no significant difference in final DSC value for any case, but with slightly higher variability for the iPlan^®^ method. To discriminate between the two DIR modalities a two-tailed Student's *t*-test at 95% CL was calculated as well as its corresponding *P*-value, obtaining *t* = 0.28 and *P* = 0.78, showing as expected no statistical differences between the two algorithm results.

**Fig. 3.**
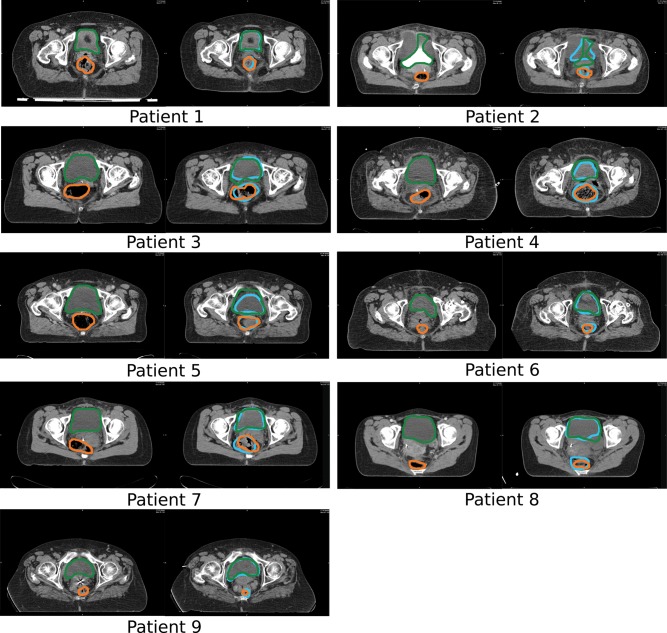
Representative axial slices from planning CT (left) and repeated CT (right) for all patients. The manually drawn bladder (green) and rectum (orange) are displayed on the planning CT. The manually contoured structures (blue) are shown in the repeated CT, together with the original planning CT structures (green and orange respectively) deformed by the DIR algorithm.

Finally in Fig. 3, an illustrative example is given that shows the registration results for a representative axial slice of the volume data for each of the patients when using the iPlan^®^ method. For each patient, on the left the original planning CT slice is presented with delineated contours (bladder in green, rectum in orange), and on the right a slice from the repeated CT with manual contours (both in blue) is shown, with the propagated contours in the same color as on the planning CT.

## DISCUSSION

In most advanced radiotherapy approaches inverse planning or computerized optimization approaches are used for treatment planning, which are driven by tolerance doses to OAR. Accurate OAR tracking in repeated images is thus considered to be essential in advanced radiotherapy that is based on the delivery of steep-dose gradients. Image-guided radiotherapy (IGRT) and ART approaches have been proposed to study and manage interfraction deformation, and to take action in case significant anatomical changes occur. DIR is generally considered as being a prerequisite for contour propagation in a time-efficient manner, since it eliminates the need for workload-intensive manual contouring. Furthermore DIR is needed for dose-accumulation approaches. Dose accumulation is, however, not the primary focus of the present study. Therefore, it is crucial to investigate the validity of DIR for each tumour site and image modality [17, 18].

In the current study DIR was applied to propagate OAR volumes for cervical cancer patients undergoing external beam therapy, i.e. planning CTs were compared to images acquired during external beam therapy. As a golden standard, deformed contours/volumes were compared with manually drawn contours, which were delineated directly on repeated imaging datasets. Neither of the methods used revealed a significant improvement in volume overlap when propagating contours from planning CT to repeated images. The results obtained were similar for both organs and were independent of the initial volume overlap, showing no particular improvement or decrease of overlap after RR compared with the initial overlap, indicating that independently of the amount of organ deformation there is not enough information in the images to correctly extract a meaningful deformation field using the DIR methods compared in this paper. This deformation field cannot, therefore, be used to propagate contours. Additional and extended studies, including other methods or improved versions of these ones, are needed in order to conclude whether or not DIR can be used successfully in the pelvic region, as previously demonstrated for other regions [19].

Possible reasons for failure might be the high-intensity variations caused by the presence of air and the very large deformations observed in the bladder for some patients. In our study, patients were instructed to have a ‘comfortably filled bladder’ both during image acquisition for treatment planning and therapy. The disadvantage of this approach is that it is up to the patients to establish the filling, and this can lead to large variations in the actual bladder volume. Nevertheless, establishing a quantitative filling approach is also challenging and can also result in significant volume changes.

In some cases, even if the *DSC* values did not show good agreement between the DIR propagated contours and the manually defined contours, deformation was following the correct trend in terms of shape for some regions. This is illustrated in Figure 3. But it is also clear that the DIR algorithms cannot cope with the larger deformations along other directions, especially bladder deformations along the cranial-caudal direction, which could be seen on sagittal plane images. Further investigation is necessary to draw final conclusions, and to be able to work on improvements on the DIR algorithm for this tumour site.

Most interestingly, even though the amount of deformation achieved with DIR for each structure (propagated contour) was not high, results obtained for CT-CT and CT-CBCT registration were following similar deformation trends, i.e. the investigated DIR algorithms did not depend on whether inter- or intra-modality registration was performed. This could not be expected *a priori*, since CBCT is known to have impaired image quality. Repeated imaging for ART is a demanding procedure in terms of workload. If imaging during the course of radiotherapy is performed with a CT, patients have to be separately imaged. CBCT technology available on state-of-the-art medical LINACs are advantageous in this respect, as imaging can be done simply prior to the delivery of a daily fraction, with no special demands on the logistics. Our current study and the previous study [20] underline the value of CBCT for tracking the position of OARs, even in the pelvic region. The limited soft tissue contrast of CBCT is still high enough to manually delineate OARs such as the bladder or rectum, and most importantly may also be able to to drive DIR algorithms, as shown in the present study.

Monitoring interfraction OAR variation is important in IGRT and ART, and achievable with CBCT technology in many treatment sites. However, in ART this needs to be completed by assessing the target position and/or variations, respectively. CBCT imaging quality can be considered to be inadequate for this step. This step requires CT or MR, which is the current standard for target definition. Multimodality imaging is known to improve intra-observer variations in target definition and opens the gate towards a biologically driven target concept [21]. In the light of these considerations one might ask whether there is room for CBCT-guided ART? Biological changes (changes in perfusion status, hypoxia) in the target, or tumour shrinkage as response to therapy, usually occur with larger time scales than filling variations in OARs. Thus it is a reasonable approach to use imaging techniques like CT or MR once or twice during the course of radiotherapy to assess target changes, and CBCT to track OAR variations. The integration of all this imaging information into an ART protocol needs to be tackled in the future. Obtaining adequate performance of DIR for CBCT images is an important step for ART concepts in the pelvic regions.

## CONCLUSION

In conclusion, both the performance of our in-house developed featurelet-based DIR and the iPlan^®^ TPS DIR algorithms is not accurate enough—for the time being—for automatic contour propagation in the pelvic region. Further development is needed to utilize the potential of DIR for ART in this anatomic region. The results obtained for contour propagation of bladder and rectum were comparable to the results obtained by other groups [22, 23].

## FUNDING

This work was supported by Marie Curie Actions, Particle Training Network for European Radiotherapy (PARTNER) (Grant Agreement No. 215840) and EBG MedAustron GmbH. The financial support by the Federal Ministry of Economy, Family and Youth and the National Foundation for Research, Technology and Development is gratefully acknowledged.
